# Assessing the performance of Granger–Geweke causality: Benchmark dataset and simulation framework

**DOI:** 10.1016/j.dib.2018.10.034

**Published:** 2018-10-16

**Authors:** Mattia F. Pagnotta, Mukesh Dhamala, Gijs Plomp

**Affiliations:** a Perceptual Networks Group, Department of Psychology, University of Fribourg, Fribourg CH-1701, Switzerland; b Department of Physics and Astronomy, Georgia State University, Atlanta, GA 30303, USA; c Neuroscience Institute, Georgia State University, Atlanta, GA 30303, USA

**Keywords:** Additive noise, Barrel cortex, Brain connectivity, Common reference problem, Conditional Granger causality, EEG, Nonparametric Granger causality, SNR imbalance

## Abstract

Nonparametric methods based on spectral factorization offer well validated tools for estimating spectral measures of causality, called Granger–Geweke Causality (GGC). In Pagnotta et al. (2018) [1] we benchmarked nonparametric GGC methods using EEG data recorded during unilateral whisker stimulations in ten rats; here, we include detailed information about the benchmark dataset. In addition, we provide codes for estimating nonparametric GGC and a simulation framework to evaluate the effects on GGC analyses of potential problems, such as the common reference problem, signal-to-noise ratio (SNR) differences between channels, and the presence of additive noise. We focus on nonparametric methods here, but these issues also affect parametric methods, which can be tested in our framework as well. Our examples allow showing that time reversal testing for GGC (tr-GGC) mitigates the detrimental effects due to SNR imbalance and presence of mixed additive noise, and illustrate that, when using a common reference, tr-GGC unambiguously detects the causal influence׳s dominant spectral component, irrespective of the characteristics of the common reference signal. Finally, one of our simulations provides an example that nonparametric methods can overcome a pitfall associated with the implementation of conditional GGC in traditional parametric methods.

**Specifications table**

**Table:** 

Subject area	*Neuroscience*
More specific subject area	*Granger-causal analysis*
Type of data	*Benchmark dataset: rats epicranial EEG recordings*
	*Simulation framework: numerical simulations*
How data was acquired	*Benchmark dataset: arrays of 16 stainless steel electrodes*
Data format	*Benchmark dataset: MATLAB files (.mat)*
	*Simulation framework: codes and functions (.m) implemented in MATLAB® (The MathWorks, Inc.)*
Experimental factors	*Benchmark dataset: signals were sampled at 2 kHz and online filtered 1–500 Hz*
Experimental features	*Benchmark dataset: multichannel somatosensory evoked potentials (SEPs) recorded from ten p21 Wistar rats during unilateral whisker stimulations; the animals were under light isoflurane anesthesia while recording*
Data source location	*Fribourg, Switzerland*
Data accessibility	*Benchmark dataset, simulation framework and codes for estimating nonparametric Granger causality are made available with this article.*
Related research article	*M.F. Pagnotta, M. Dhamala, G. Plomp, Benchmarking nonparametric Granger causality: robustness against downsampling and influence of spectral decomposition parameters, NeuroImage. 183 (2018) 478–494.*https://doi.org/10.1016/j.neuroimage.2018.07.046

**Value of the data**

• Provides information about a benchmark dataset that allows to critically assessing the performance of time-varying directed connectivity measures.
• The simulation framework enables the readers to evaluate the effects of common practical issues associated with the application of GGC analyses.
• Makes available scripts that can be used for computing nonparametric GGC.
• Demonstrates that nonparametric methods overcome the issues due to model subset of conditional GGC.

## 1 Data

The benchmark dataset includes multichannel epicranial somatosensory evoked potentials (SEPs) recorded from ten rats during whisker stimulations. Since in this animal model both physiological characteristics and structural pathways have been intensively investigated, reliable predictions can be made about the dominant cortical driver, its preferential functional targets on the cortex, and the timing off such directed interactions. For these reason, this dataset represents a valuable tool to evaluate the performance of time-varying directed connectivity measures and compare different algorithms for connectivity analysis.

Here, we also provide codes and functions to perform a series of simulations that can evaluate the effects of some practical issues associated with the application of GGC analyses to real data.

## 2 Experimental design, materials, and methods

### 2.1 Whisker-evoked SEPs

In the benchmark dataset, the multichannel epicranial SEPs have been previously obtained from ten p21 Wistar rats [2], [3]. Signals were recorded using an array of 16 stainless steel electrodes positioned on the cranium [3]. Among them, 15 electrodes acquired epicranial SEPs, and these were referenced to a reference electrode (16th electrode), which was placed above the cerebellum, as shown in Fig. 1A in [1]. Signals acquisition was performed at sampling rate of 2000 Hz and the signals were filtered online with a bandpass filter (1–500 Hz). During the experimental procedure the rats were anesthetized using isoflurane in air mixture (oxygen/air: 20%/80%). The stimuli consisted of unilateral whisker deflections of 500 μm, which were applied using a solenoid-based stimulator. For each animal, 50 stimulations were applied to right whiskers and 50 to left whiskers, in two separate blocks. Animal handling procedures were approved by the Office Vétérinaire Cantonal (Geneva, Switzerland) in accordance with Swiss Federal Laws.

**Fig. 1:**
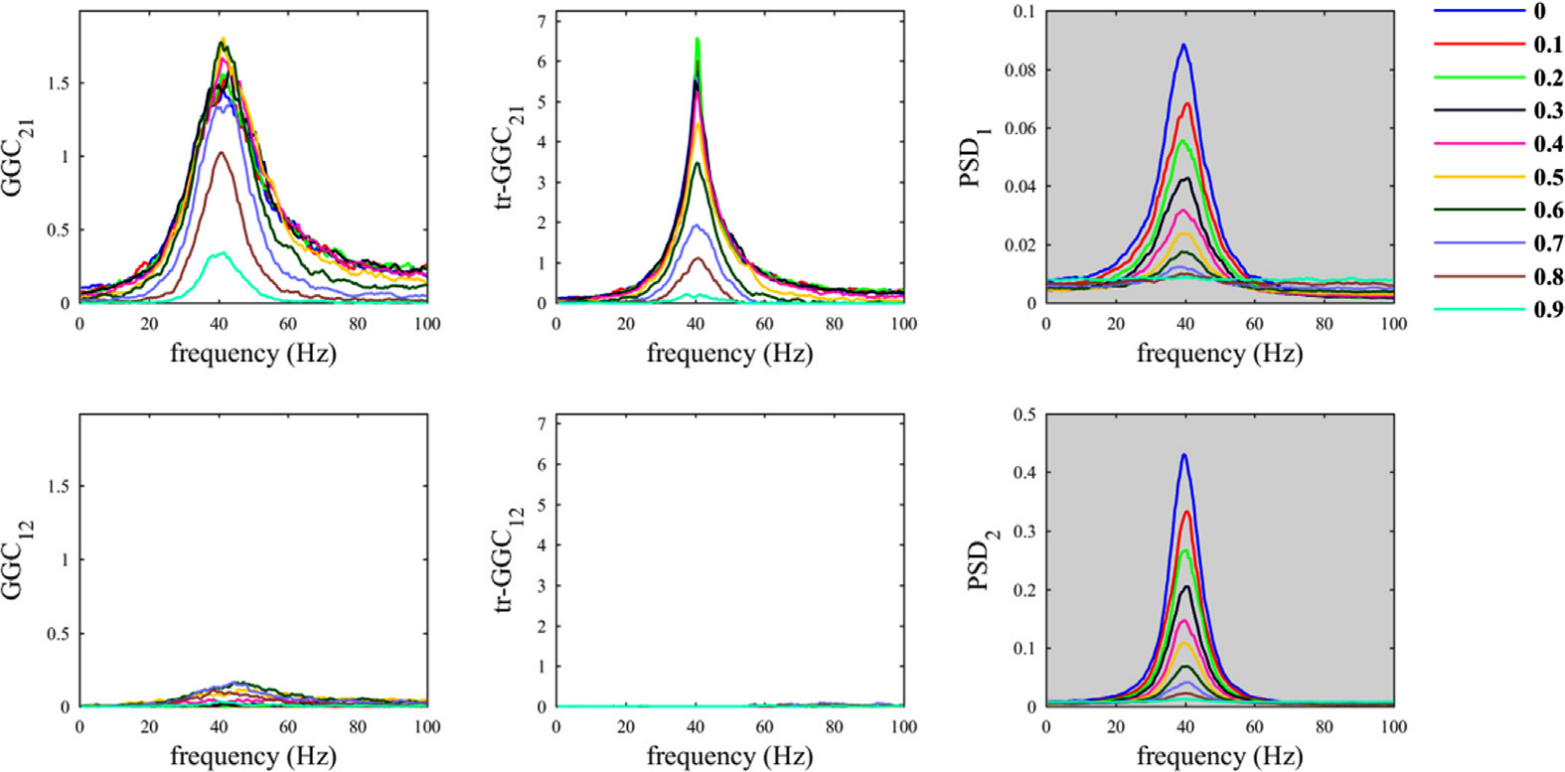
Common reference problem: scenario where the common reference *R(t)* is an uncorrelated white noise.

We successively defined trials considering the time interval between -100 ms and +200 ms around each stimulus onset. We then applied a semi-automatic approach to remove trials corrupted by artifacts; more details about this approach and a comprehensive list of trials removed can be found in [4] and its supporting information. Survived trials from the two blocks were then collected together, by changing electrodes labels in the case of left-sided stimulations, so that electrodes from 1 to 7 were on the hemisphere ipsilateral to whisker stimulation and electrodes from 9 to 15 were on the contralateral one for both blocks. The amount of survived trials for each animal was on average 65 (range: 34–80).

The dataset is made available with this article and comprises ten *.mat* files (one for each animal), which were created using MATLAB® (The MathWorks, Inc.). Each file contains a structure named *RAT* with four fields: *data*, *dimord*, *times*, and *Fs*. The field *data* is a 3-dimensional matrix with the epicranial SEPs, being the time-points on the first dimension, the channels on the second dimension, and the trials on the third dimension. Dimensionality ordering is also specified by the field *dimord*, which is a string. The field *time* contains a vector of time-points in milliseconds. Finally, the field *Fs* is a scalar that reports the sampling rate in Hz.

Whisker-evoked SEPs are characterized by highly dynamic cortical activation pattern, which is initially observable over contralateral primary sensory cortex (cS1), and then propagate over known areas, following relatively well known structural pathways [5], [6]. The latencies of this propagation are also known, thank to studies that employed single-unit responses in cS1, e.g. [7]. The functional characteristics expected in the cortical network of 15 nodes allow benchmarking time-varying directed connectivity measures using three previously defined performance criteria [2], which evaluate the ability of a method to detect cS1 as main functional driver, and the contralateral parietal and the frontal sensory-motor cortex as cS1׳s preferential targets. In the dataset, channel 12 identifies cS1, while channels 10 and 14 identify the surrounding portions of cortex, which are expected to be cS1׳s preferential targets. For a schematic representation of channels positions please refer to Fig. 1A in [1]. The initial driving from cS1 is expected at early latency after stimulus onset (5–20 ms), and it should be characterized by dominant spectral components in the gamma-band [8], [9], [10].

### 2.2 Simulation framework

Previous studies have highlighted a series of pitfalls and practical issues associated with the use of causality analyses in real data. We here provide a simulation framework that enables the reader to evaluate three of these issues: the common reference problem [11], the imbalance of signal-to-noise ratio (SNR) between channels [12], and the effects of independent and mixed additive noise [13]. Moreover, we provide a numerical simulation that allows to address a recent claim of pitfall [14] associated with the use of conditional GGC [15], [16]. All simulations employ nonparametric GGC methods based on spectral factorization [17], [18].

Our simulation framework comprises three scripts implemented in MATLAB® (The MathWorks, Inc.): i) *sim_nonparGGC_CommonReference.m*, which allows evaluating the common reference problem; ii) *sim_nonparGGC_AdditiveNoise.m*, which allows assessing the effects of SNR differences and presence of additive noise; iii) *sim_nonparGGC_StokesPurdon.m*, which addresses the claim of pitfall of conditional GGC.

In the first two scripts, both traditional and time-reversed definitions of GGC are considered, because time reversal testing for causality analyses [19], [20] has been shown to potentially reduce, or at least alleviate, some of the detrimental effects associated with the mentioned practical issues. In this procedure, time-reversed versions of the time series are used as surrogates for statistical testing. Different definitions have been proposed for the time-reversed variant of GGC (tr-GGC), we here choose by default the “difference-based” one, in which the spectral influence from channel *j* to channel *i* tested with time-reversal (*tr-GGCij*) is defined as the difference between the *net-GGCij* estimated on regular time series and the same measure estimated on time-reversed time series [21]. Each measure of *net-GGCij* is obtained in turn as difference between *GGCij* and *GGCji*. The net influence *tr-GGCij* is finally inferred only when larger than zero (see Eq. (6) in [1]). The alternative time-reversed variants are also available in the scripts. As default settings for our simulations, time series are simulated with 100 trials for each condition evaluated. Each trial has a length of 2 s, corresponding to 400 samples at sampling frequency Fs = 200 Hz. In all conditions nonparametric GGC is computed with the approach based on multitaper method [22], by selecting the time-bandwidth parameter *NW* = 4 [23]; for further details see [1]. The spectral estimation is performed up to Nyquist frequency.

The script *sim_nonparGGC_StokesPurdon.m* allows simulating the same MVAR(3) three-nodes network used in [14], and successively applying the multitaper-based conditional GGC to the simulated data.

#### 2.2.1 Common reference problem

The common reference problem involves a situation where the signals from the nodes of the network are recorded against a common reference, which is not electrically silent. This may have detrimental effects on functional connectivity measures. The script *sim_nonparGGC_CommonReference.m* allows to consider three main scenarios, depending on the characteristics of the common reference signal *R(t)*: i) *R(t)* is simulated as uncorrelated white noise, as in [11]; ii) *R(t)* has an oscillatory component in the same frequency range as the investigated nodes, which is similar to the situation considered in [12]; iii) *R(t)* has an oscillatory component in a different frequency range with respect to the investigated nodes.

In order to simulate the model, we start considering a basic surrogate network that is composed by two nodes with a causal influence between them (from node 1 to node 2). The unipolar signals of the two nodes are obtained from a MVAR model with maximum lag of 2 time samples, using the following equations:

(1) $$\{\begin{array}{l} x_{1}\left(t\right)\;=\;2r_{1}\cos{(\theta }_{1})x_{1}\left(t-1\right)\;-{\;r_{1}}^{2}x_{1}\left(t-2\right)\;+\;w_{1}(t)\\ x_{2}\left(t\right)\;=\;{2r_{2}\cos{(\theta }_{2})x}_{2}\left(t-1\right)\;-{\;r_{2}}^{2}x_{2}\left(t-2\right)\;-{\;0.35x_{1}\left(t-1\right)\;+\;0.7x_{1}\left(t-2\right)\;+\;w}_{2}(t) \end{array}$$

As default, we set *r*_*i*_ = 0.8 for *i* = 1,2. The parameter *θ*_*i*_ is defined as *θ*_*i*_ = *2πf*_*i*_*Δt*, where *Δt* is the inverse of the sampling frequency and *f*_*i*_ is the desired frequency of the oscillatory component. We set *f*_*i*_ = 40 Hz for *i* = 1,2, in this way the dominant oscillatory component of both nodes is around 40 Hz [24]. In Eq. (1), each term *w*_*i*_ is a zero-mean uncorrelated white noise process, called innovation process, which has an effect on the future samples of the signal *x*_*i*_ trough the coefficients of the MVAR model. This should not be confounded with the additive noise, which is sometimes also called observation noise or measurement noise. The additive noise is in fact superimposed to the measurement of the time series, i.e. the values simultaneously observed/recorded (see 2.2.2, 2.2.3).

In the first scenario, the signal *R(t)* consists of white noise with same variance as *w*_*i*_. In the second and third scenarios, *R(t)* is defined by the following equation:

(2) $$R\left(t\right)\;=\;2r_{R}\cos(\theta_{R})R\left(t-1\right)\;-{\;r_{R}}^{2}R\left(t-2\right)\;+\;w_{R}(t)$$

where *r*_*R*_ = 0.8 and *θ*_*R*_ = *2πf*_*R*_*Δt*. We use *f*_*R*_ = 40 Hz, which is the same oscillatory component as the two nodes, in order to simulate scenario ii). While for scenario iii), we use *f*_*R*_ = 20 Hz and *f*_*R*_ = 70 Hz, which are respectively below and above the oscillatory component of the two nodes in the network (40 Hz). In each simulated condition, the observed (measured) time series of each node is finally obtained as follows:

(3) $$x_{i}^{\prime }\left(t\right)\;=\;(1-\alpha_{\mathrm{CR}})x_{i}(t)\;-\;\alpha_{\mathrm{CR}}R\left(t\right),\;i=1,2$$

In this way the ratio *α*_*CR*_/(1–*α*_*CR*_) controls the proportion in the observed signals between common reference and unipolar signals, i.e. how strong the influence of the common reference is. In the simulation we vary *α*_*CR*_ in the range [0.1, 0.9] with a resolution of 0.1 (9 levels in total).

The script enables the reader to perform a simulation for each one of the three possible scenarios with respect to the characteristics of the common reference signal, by simply varying the appropriate flag-variable (i.e., *flg_REFtype*).

#### 2.2.2 SNR imbalance between channels

Interpretational problems associated with causal estimates have been observed in the presence of SNR differences across experimental conditions or between sources/channels [12], [25]. The script *sim_nonparGGC_AdditiveNoise.m* allows investigating SNR imbalance effects on nonparametric GGC, when we set the flag-variable *flg_Analysis* to *׳MIX׳*; this simulation enables to employ the same 2-nodes MVAR(2) model previously used in [12], which is defined as follows:

(4) $$\{\begin{array}{l} x_{1}\left(t\right)\;=\;0.5x_{1}\left(t-1\right)\;-\;0.8x_{1}\left(t-2\right)\;+\;w_{1}(t)\\ x_{2}\left(t\right)\;=\;0.5x_{2}\left(t-1\right)\;-\;0.8x_{2}\left(t-2\right)\;+\;{0.2x_{1}\left(t-1\right)\;-\;0.1x_{1}\left(t-2\right)\;+\;w}_{2}(t) \end{array}$$

As in Eq. (1), the terms *w*_*i*_ are zero-mean uncorrelated white noise processes. An additive noise *N*_*1*_*(t)* is then simulated as zero-mean uncorrelated white noise with same variance as *w*_*i*_ and added only to the signal of node 1. We here extend the investigation provided in [12], by using an approach similar to that implemented in Eq. (3). We vary in fact the amount of the additive noise term on the measured signal from node 1, through the parameter *α*_*N*_ in Eq. (5); the measured time series are then obtained as follows:

(5) $$\{\begin{array}{l} x_{1}^{\prime }\left(t\right)\;=\;(1-\alpha_{N})x_{1}\left(t\right)\;+\;\alpha_{N}N_{1}\left(t\right)\\ x_{2}^{\prime }\left(t\right)\;=\;x_{2}\left(t\right) \end{array}$$

In this way, the SNR of node 1 is controlled by the ratio (1–*α*_*N*_)/*α*_*N*_. Differently, additive noise is not present on node 2, meaning that the SNR on this node can be considered constant and equal to infinity. We vary the parameter *α*_*N*_ using the same values previously used for *α*_*CR*_ (see Section 2.2.1) in order to range the SNR imbalance between the two nodes in the network.

The simulation can be easily extended to the multivariate case, by varying in the script the variables that define the number of nodes in the network; in this case, the time series of each node is defined by one of the two following equations:

(6) $$\begin{array}{c} x_{i}\left(t\right)\;=\;0.5x_{i}\left(t-1\right)\;-\;0.8x_{i}\left(t-2\right)\;+\;w_{i}\left(t\right)\\ \mathrm{or}\\ x_{i}\left(t\right)\;=\;0.5x_{i}\left(t-1\right)\;-\;0.8x_{i}\left(t-2\right)\;+\;{0.2x_{j}\left(t-1\right)\;-\;0.1x_{j}\left(t-2\right)\;+\;w}_{i}(t) \end{array}$$

Here the time series of node *i* (*x*_*i*_) is defined by the first expression in (6) when the node does not receive any influence, whereas *x*_*i*_ is simulated using the second expression in (6) when the node receives a causal influence from node *j*. The user can control the simulated interactions and the list of nodes where additive noise is superimposed, by changing in the script the corresponding variables (i.e., *sim_interact* and *addN_nodes*, respectively).

#### 2.2.3 Independent or mixed additive noise

One of the most common concerns in the field of causality analyses is related to the presence of additive noise and its effects on results interpretability [13], [26], [27]. We can distinguish situations in which additive noise terms are independent from situations in which the additive noise terms are a linear mixture of multiple noise sources. Both cases can be simulated from the same model [13], [27], which is implemented in the script *sim_nonparGGC_AdditiveNoise.m*. If we consider *M* channels (nodes) and *S* independent noise sources, the additive noise model obtained with mixing can be expressed for each time instant *t* as:

(7) $$E\left(t\right)\;=\left(\begin{array}{c} e_{1}(t)\\ \vdots \\ e_{M}(t) \end{array}\right)=\;K\eta (t)\;$$

where *e*_*i*_*(t)* is the additive noise of node *i*, *K* is a linear mixing matrix of dimension *M*-by-*S*, *η(t)* is a vector of independent noise sources of length *S*. By default, in our simulation *M* = 3 and we consider the case *S*=3 for the additive noise model (Eq. (7)). We can then simulate independent additive noise by imposing *K*=*I*_*MxM*_, which is the identity matrix of dimension *M*-by-*M* (when the flag-variable *flg_Analysis* is set to *׳IND׳*). Differently, when we set the flag-variable *flg_Analysis* to *׳MIX’*, we simulate mixed additive noise by defining *K* as random matrix with full rank, which allows for example to mix independent white noise sources. Alternatively, the same simulation can also be performed considering white and pink noise sources instead of purely white [27], by changing the appropriate variable in the script (i.e., *flg_Ncolor*). In every simulation the observed time series with superimposed additive noise is then obtained as:

(8) $$X^{\prime }\left(t\right)\;=\left(\begin{array}{c} x_{1}^{\prime }(t)\\ \vdots \\ x_{M}^{\prime }(t) \end{array}\right)=\;(1-\alpha_{N})X\left(t\right)\;+\;\alpha_{N}E\left(t\right)$$

In such way, the SNR is proportional to the ratio (1–*α*_*N*_)/*α*_*N*_, and we vary the parameter *α*_*N*_ as in the previous simulations (see 2.2.1, 2.2.2).

#### 2.2.4 Simulation of Stokes and Purdon׳s example

The script *sim_nonparGGC_StokesPurdon.m* implements the MVAR(3) three-nodes network previously used in [14]; the explicit equation that defines the system can be found in the original work. In this surrogate network node 2 is driven by node 1 and node 3 is driven by node 2. The three nodes resonate at different frequencies: 40 Hz, 10 Hz and 50 Hz, from node 1 to node 3, respectively. As in the original work, we consider sampling frequency Fs = 120 Hz and simulate 1000 realizations with length of 500 samples each. We employ the multitaper-based approach to estimate nonparametric GGC, selecting *NW* = 4 by default. This simulation allows demonstrating that nonparametric methods can overcome the pitfall associated with the implementation of conditional (full-multivariate) GGC in traditional parametric methods, which originates from fitting separately full and reduced MVAR models.

## 3 Influence of practical issues on nonparametric GGC: some examples

### 3.1 Common reference problem

The script *sim_nonparGGC_CommonReference.m* allows evaluating the effects on causality measures of the use of a common reference. We first considered the case in which the common reference signal is white noise and we observed a decrease in GGC estimates values for the true imposed influence (from 1 to 2), in both traditional and time-reversed variants by increasing the parameter *α*_*CR*_ (Fig. 1). On the other hand, *GGC*_*12*_ estimates values tended to increase when increasing parameter *α*_*CR*_. The latter were de facto spurious estimates. Differently, for *tr-GGC*_*12*_ we observed null estimates around 40 Hz irrespective of the level of the parameter *α*_*CR*_, and consequently we had unambiguous detection of the directionality of the 40 Hz causal influence in this range; while, some spurious estimates were obtained at lower and higher frequencies.

**Fig. 2:**
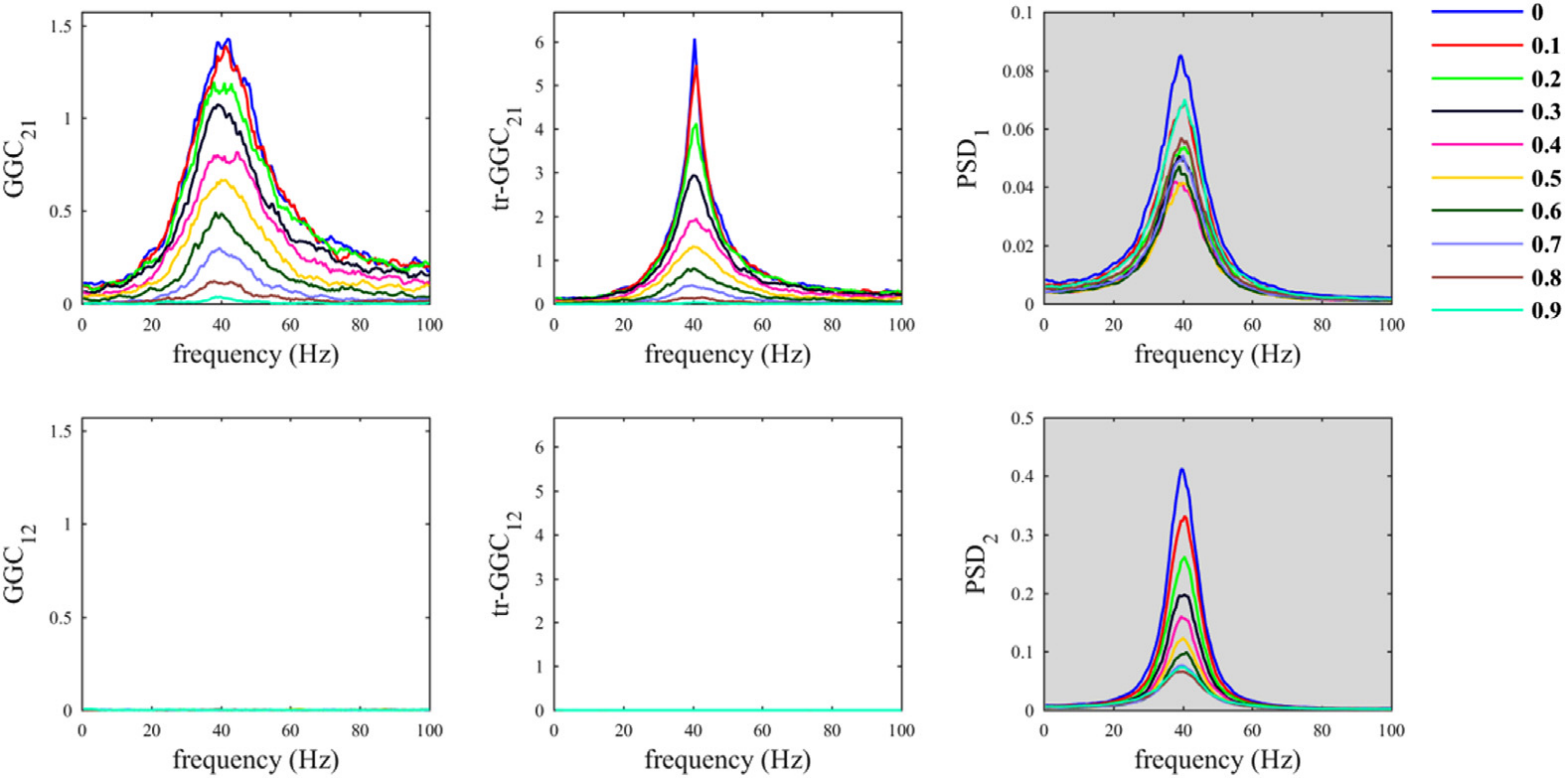
Common reference problem: scenario where the common reference *R(t)* has the same oscillatory component of the nodes.

The situation where the common reference had the same oscillatory component as the two nodes was less problematic (Fig. 2). As a matter of fact, despite increasing *α*_*CR*_ produced a decrease in both *GGC*_*21*_ and *tr-GGC*_*21*_ estimates values, we found almost null estimates for the causal influence from 2 to 1 in both GGC variants. This basically made the identification of the correct causal influence directionality in the network unambiguous, even considering big values for the parameter *α*_*CR*_.

**Fig. 3:**
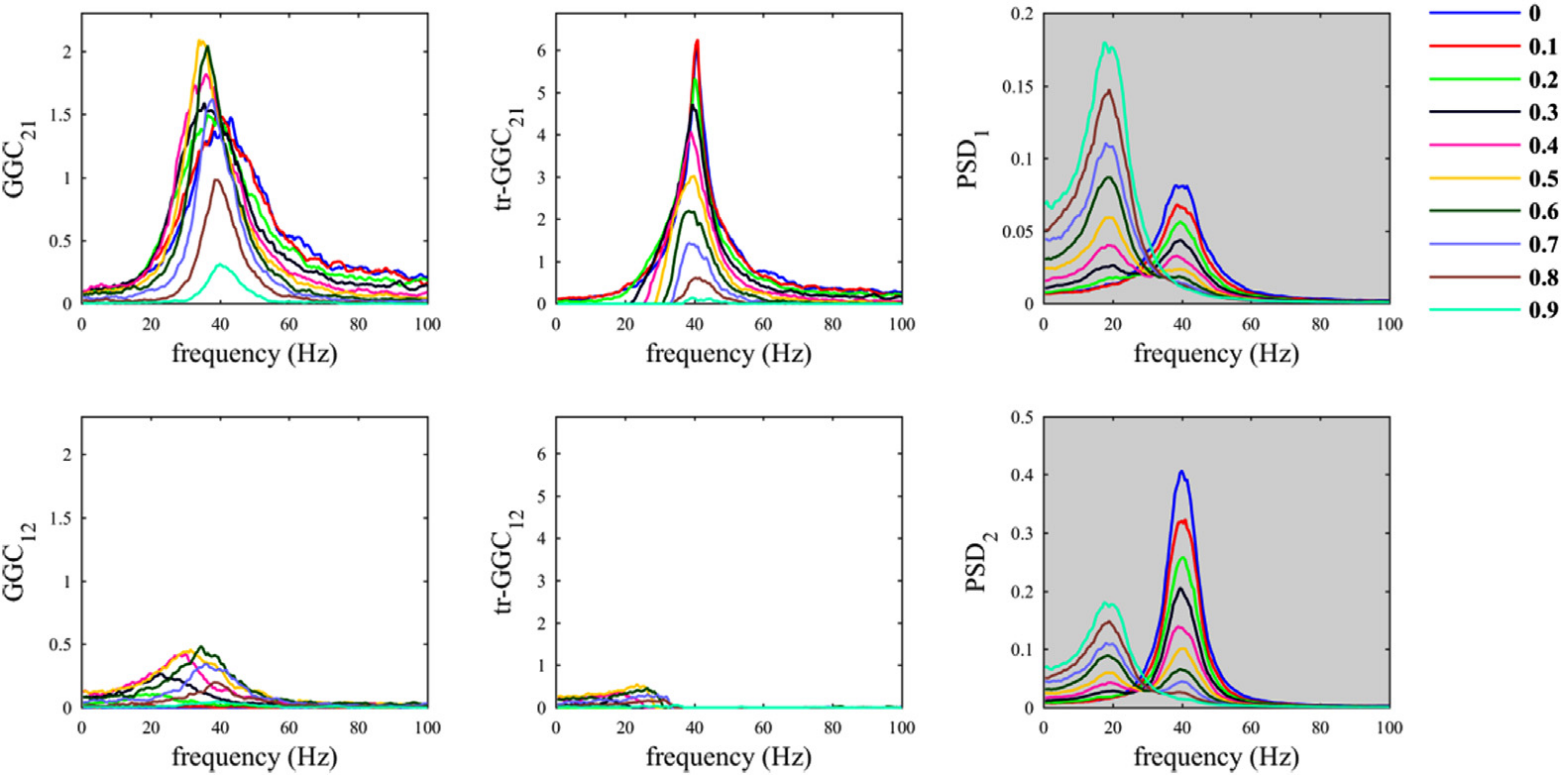
Common reference problem: scenario where the common reference *R(t)* has an oscillatory component around 20 Hz.

**Fig. 4:**
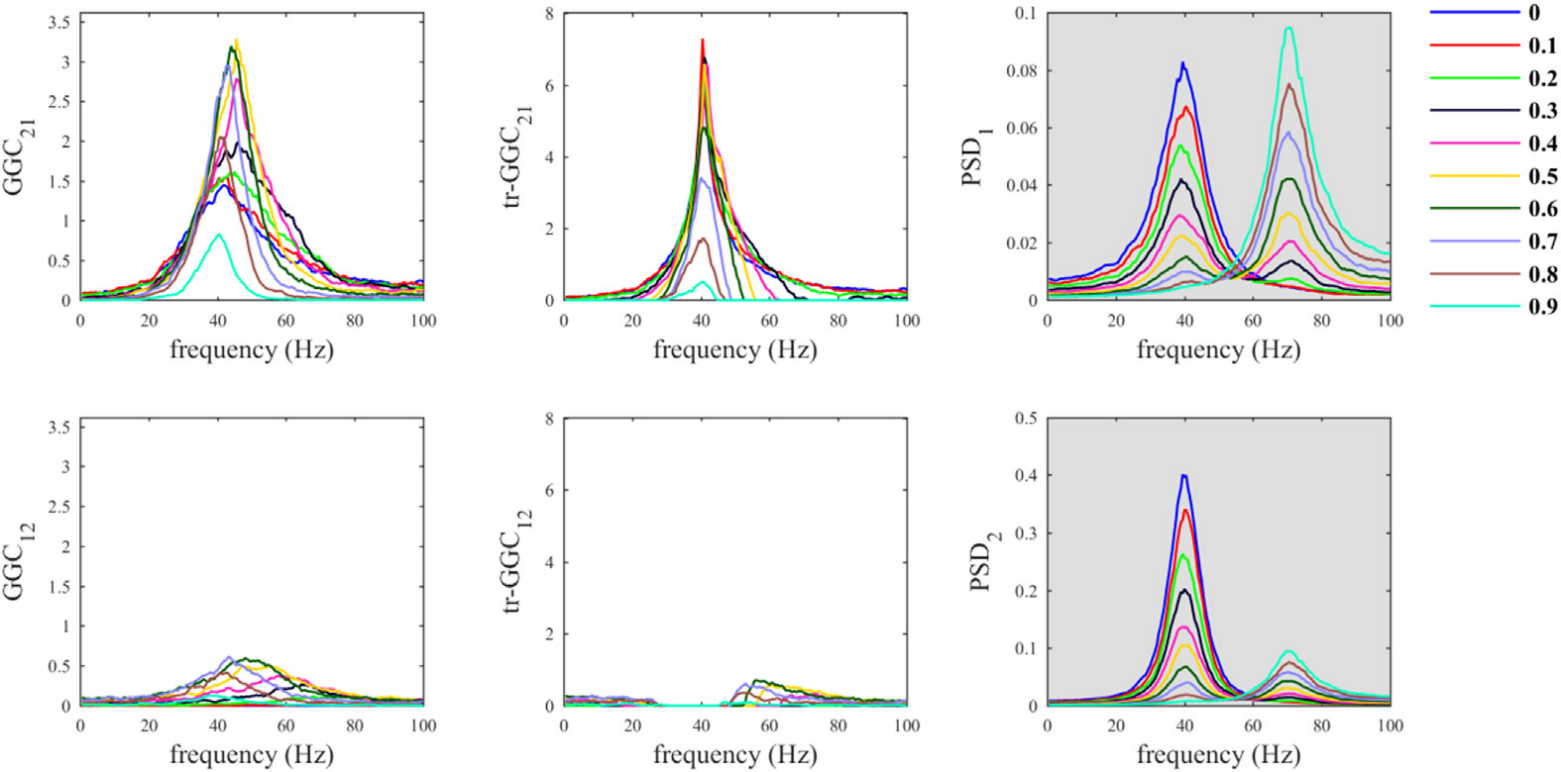
Common reference problem: scenario where the common reference *R(t)* has an oscillatory component around 70 Hz.

When the common reference was simulated with dominant oscillatory component in a different frequency range with respect to the other nodes, we observed behaviors similar to those previously obtained using white noise as common reference. In fact, only with tr-GGC we consistently obtained unambiguous detection of the 40 Hz causal influence directionality for both the 20 Hz (Fig. 3) and the 70 Hz common reference (Fig. 4). Spurious estimates were found for *GGC*_*12*_ and *tr-GGC*_*12*_; these were prevalently localized at low frequencies for the 20 Hz *R(t)*, and more biased towards high frequencies when the common reference had 70 Hz oscillatory component.

Overall, our numerical simulation allows to demonstrate that, when two interacting nodes have same dominant spectral component and the causal influence between them is unidirectional, the use of tr-GGC guarantees unambiguous detection of the causal influence directionality on frequencies around the dominant spectral component itself, regardless the characteristics of common reference signal; while, spurious estimates may occur outside such frequency-range and with a preferential frequency-space localization that depends on the spectral components of the common reference signal.

### 3.2 SNR imbalance between channels

**Fig. 5:**
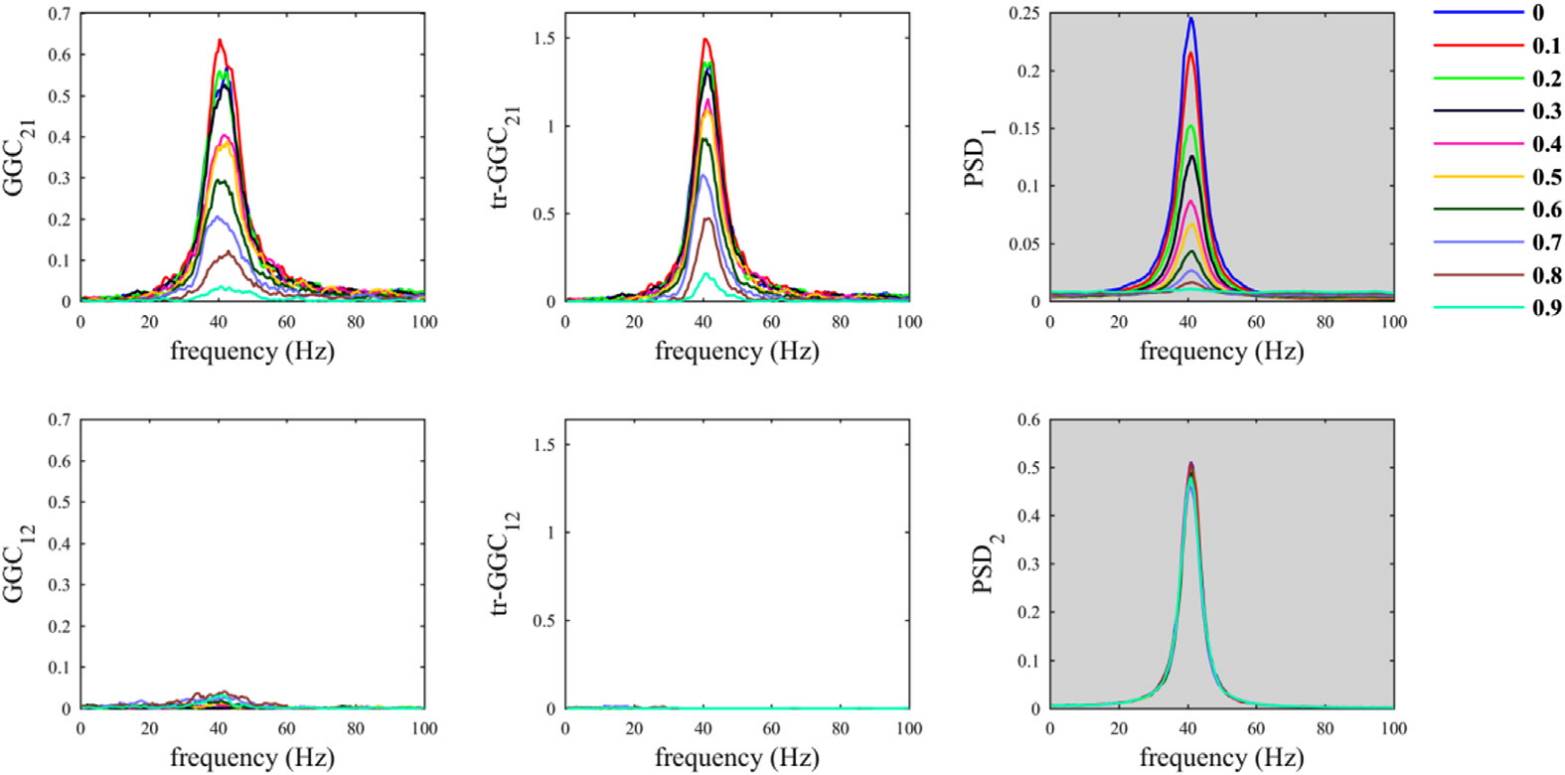
SNR imbalance between channels (bivariate case): simulation where observation noise was added only to node 1 (driver), whereas no additive noise was present on node 2 (receiver).

The script *sim_nonparGGC_AdditiveNoise.m* allows to replicate the findings about SNR imbalance from a previous study [12]. Using this simulation we also observed that increasing the parameter *α*_*N*_ produced a decrease in *GGC*_*21*_ estimates values and a contemporary increase in *GGC*_*12*_ estimates values, which are spurious (Fig. 5). Differently, despite increasing *α*_*N*_ produced a decrease in *tr-GGC*_*21*_ estimates values, the use of time reversal testing guaranteed unambiguous discrimination of the correct directionality of driving in the network. The *tr-GGC*_*21*_ estimates were in fact consistently null for frequencies around 40 Hz, and very close to zero elsewhere.

Our script permits to evaluate different situations. For example, if we still added noise only to node 1 but considered a reverse causal influence in the model, i.e. from 2 to 1 instead of from 1 to 2, the SNR imbalance problem would be negligible and we would obtain correct identification of causal directionality with both GGC and tr-GGC. Differently, if the causal influence was bidirectional with same weights in the MVAR model, we would obtain net contributes close to zero only with tr-GGC and a spurious net causal influence from node 2 to node 1 with GGC, as shown in [12].

**Fig. 6:**
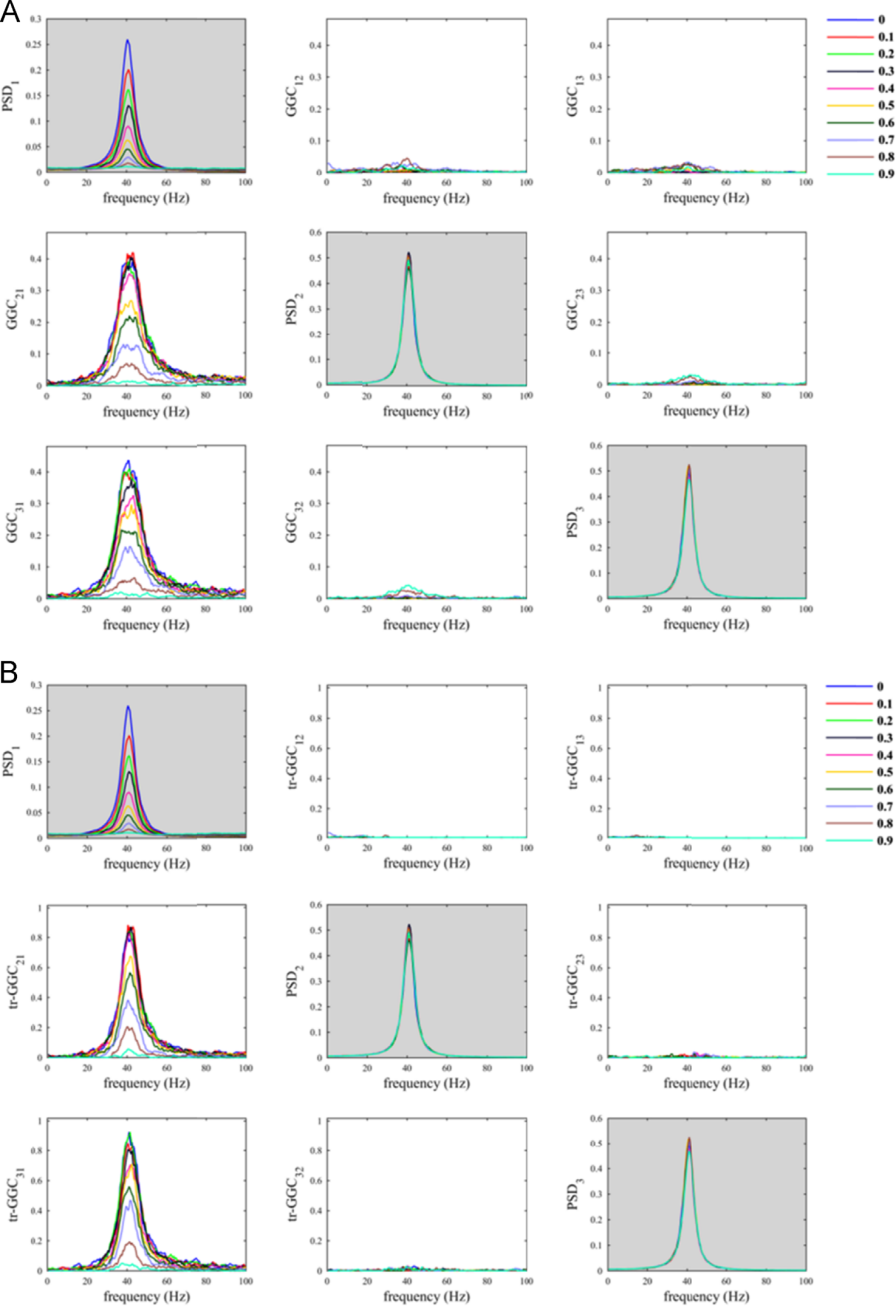
SNR imbalance between channels (trivariate case): A) conditional GGC; B) conditional tr-GGC. In this simulation we added observation noise only to node 1 (driver), whereas no additive noise was present on nodes 2 and 3 (receivers).

As mentioned above, our script enables the reader to extend the simulation to the multivariate case. In order to prove that, we considered a trivariate process in which nodes 2 and 3 received driving from node 1, according to Eq. (6). Exactly as in the bivariate case (Eq. (4)), the additive noise was superimposed only to the time series of node 1. We here employed the conditional GGC, which takes advantage of the full multivariate recordings [15], [16], [28], by setting the variable *doconditional* equal to 1 in the script. The simulation with the trivariate process confirmed that the use of tr-GGC guaranteed unambiguous discrimination of the correct driving directionalities of in the network (Fig. 6).

Conditions characterized by SNR imbalance between channels can be further complicated when using the pairwise implementation of GGC, which consist of performing repeated bivariate analyses for all combinations of channels pair. As example, we considered a trivariate process simulated using Eq. (6), where the causal influences were imposed this time from node 1 to node 2, and from node 2 to node 3; differently from the previous simulation, the additive noise was present only on node 2. We then employed the pairwise GGC for causality analysis, by setting the variable *doconditional* equal to 0 in the script.

**Fig. 7:**
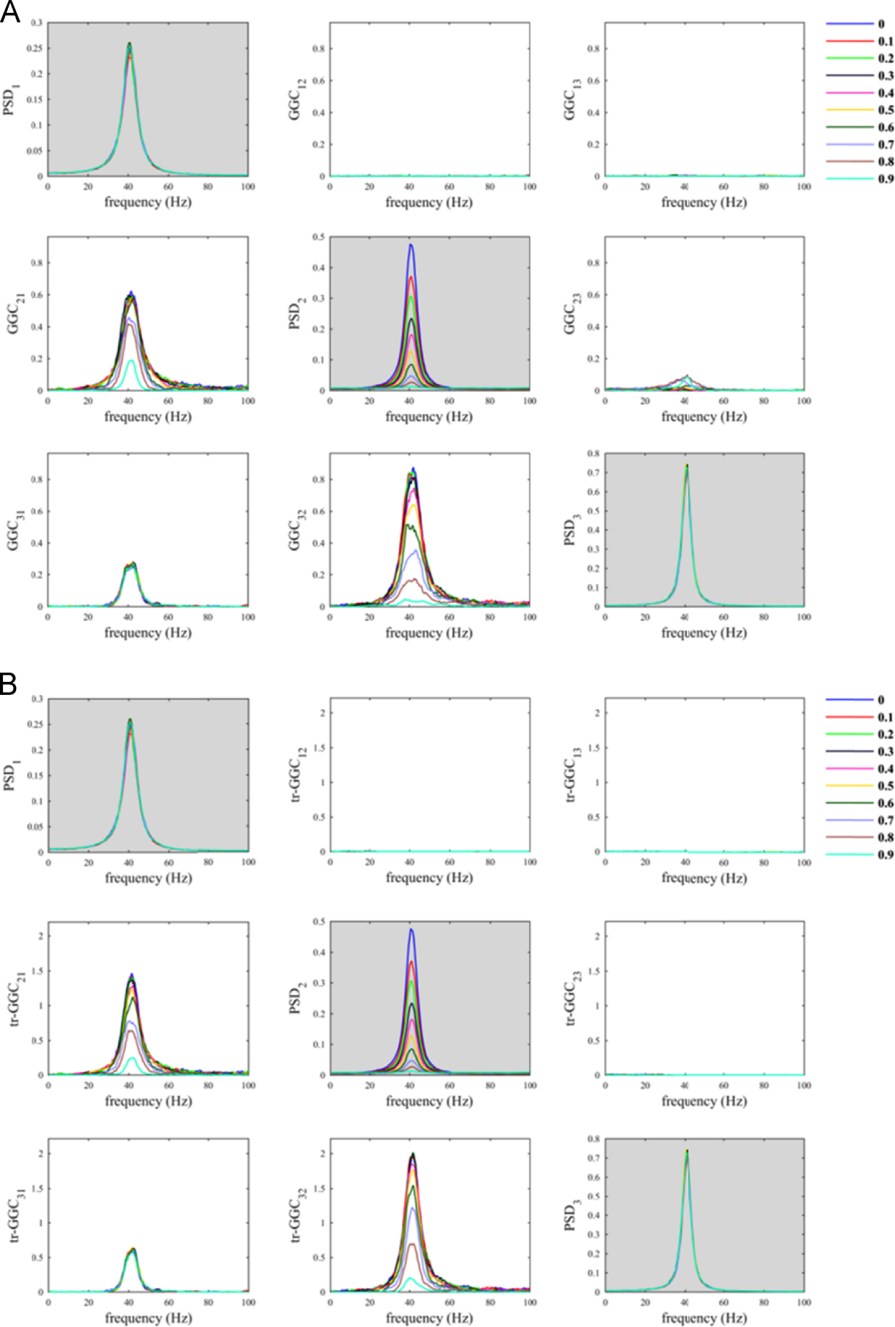
SNR imbalance between channels (trivariate case): A) pairwise GGC; B) pairwise tr-GGC. In this simulation we added observation noise only to node 2 and interactions were imposed from node 1 to node 2 and from node 2 to node 3.

When we used the pairwise definition we obtained spurious estimates values for both *GGC*_*31*_ and *tr-GGC*_*31*_ (Fig. 7), which is the typical influence due to the indirect path through node 2; interestingly these contributes were basically independent from the value of the parameter *α*_*N*_, which means that even reducing the contribute of the noise we cannot reduce these spurious estimates. For the true influences (1->2 and 2->3) the estimates values were reduced by increasing *α*_*N*_. As a consequence, when *α*_*N*_ ≥ 0.7 the spurious estimates values for the null causal influence (1->3) became comparable to those of the true influences, misleading results interpretability.

**Fig. 8:**
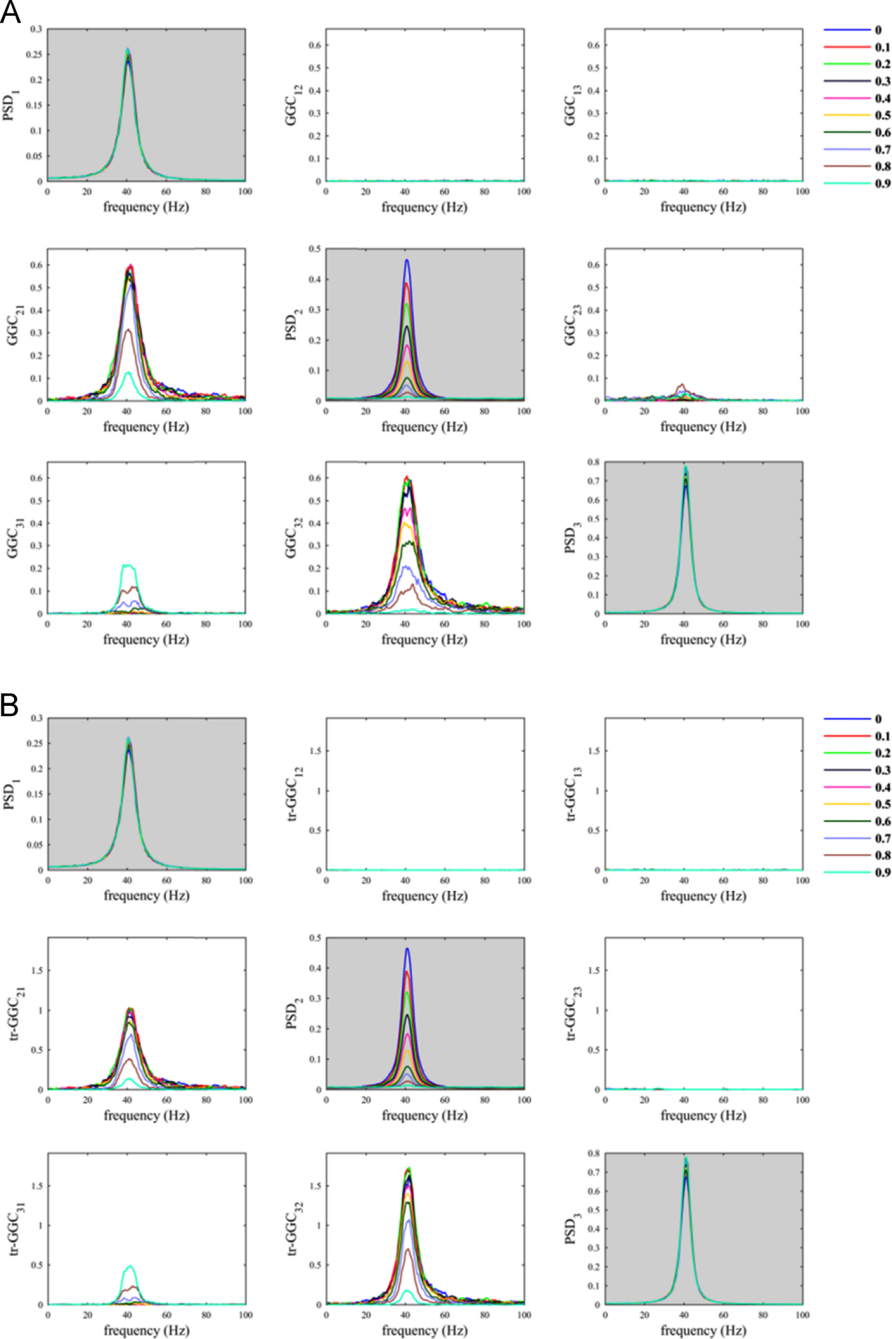
SNR imbalance between channels (trivariate case): A) conditional GGC; B) conditional tr-GGC. In this simulation we added observation noise only to node 2 and interactions were imposed from node 1 to node 2 and from node 2 to node 3 (same as in Fig. 7).

Differently, for the conditional definition we observed that *GGC*_*31*_ and *tr-GGC*_*31*_ were influenced by variations in *α*_*N*_, in such way that estimates values were reduced when decreasing *α*_*N*_. This is definitely a positive effect, because it means that reducing the noise we can suppress spurious estimates and improve results interpretability. The true influences were unambiguously distinguished when *α*_*N*_ < 0.7, because in this range the influence due to indirect path was negligible for both GGC implementations (Fig. 8). This confirm that conditional GGC should be preferred in practice over pairwise GGC [16], [17].

Overall, the use of tr-GGC helped reducing the spurious estimates on the remaining three edges with null connections, i.e. 2->1, 3->1, and 3->2, with respect to when using GGC, for both pairwise (Fig. 7) and conditional (Fig. 8) definitions.

### 3.3 Additive noise

We employed the script *sim_nonparGGC_AdditiveNoise.m* to evaluate the influence of independent additive noise on nonparametric GGC. Here, time series were simulated using the same trivariate process previously used to evaluate the difference between pairwise and conditional GGC (Section 3.2, Fig. 7, Fig. 8). Causal influences were imposed from node 1 to node 2 and from node 2 to node 3, using the expressions from Eq. (6).

**Fig. 9:**
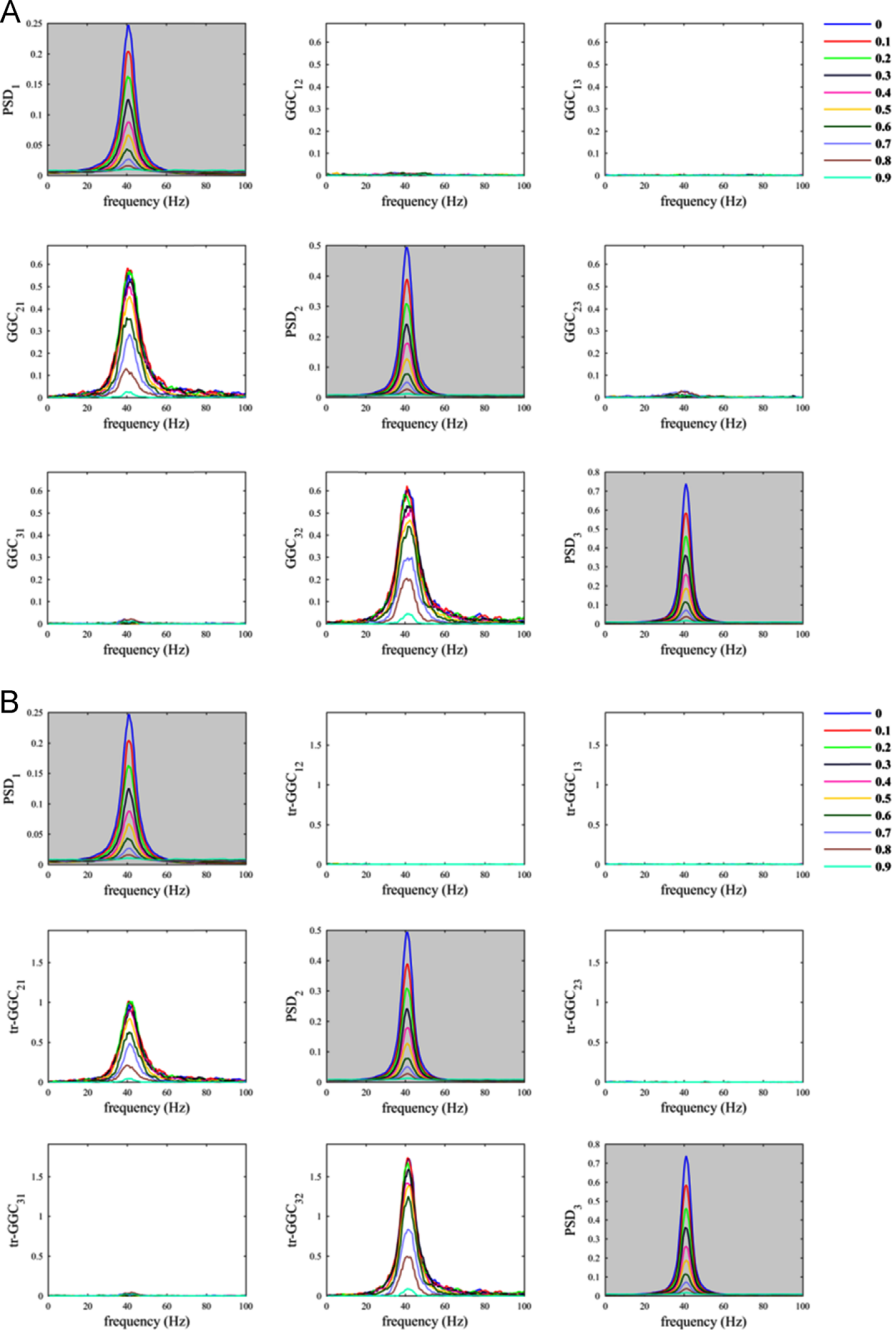
Independent white noise: A) conditional GGC; B) conditional tr-GGC. In this simulation we added independent uncorrelated white noise to both nodes.

While we observed an overall reduction in estimates values for the true causal influences when reducing SNR, we obtained also a reliable identification of the correct directions in the network (Fig. 9), confirming that the presence of independent white noise do not strongly influence the interpretability of the results obtained with causality analyses [13], [21].

**Fig. 10:**
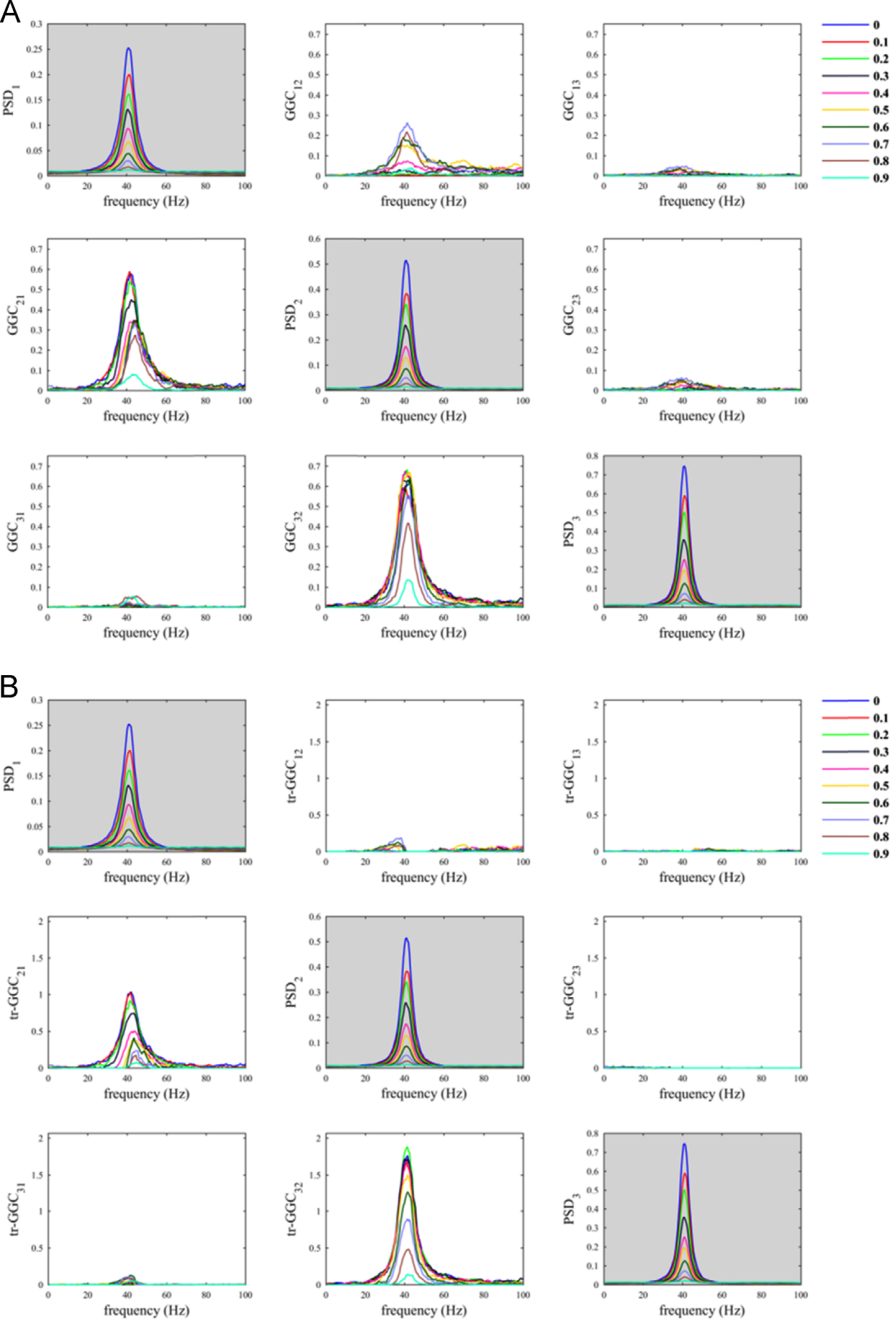
Mixed white noise: A) conditional GGC; B) conditional tr-GGC. Here the additive noise was simulated as mixing of white noise sources.

**Fig. 11:**
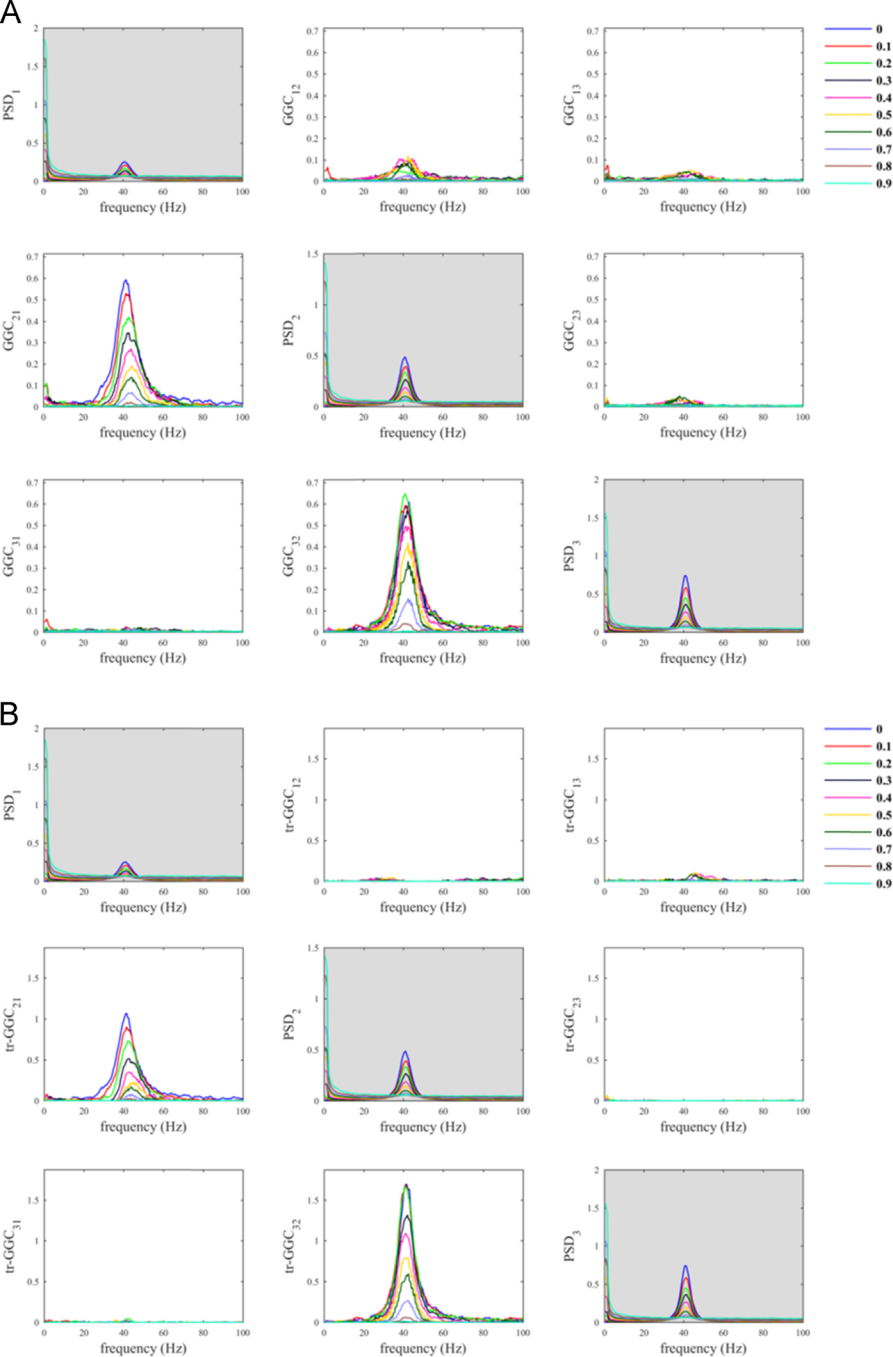
Mixed white and pink noise: A) conditional GGC; B) conditional tr-GGC. Here the additive noise was simulated as mixing of pink and white noise sources.

In a different way, the presence of mixed noise can produce detrimental effects on results interpretability. In the example here considered, we observed in fact an increase in estimates values for null influences using GGC (Fig. 10A). Such increase in spurious estimates came also with increased estimates variability, especially when we considered the case of mixed white and pink noise sources (Fig. 11A). In general, time reversal testing helped mitigating these negative effects (Figs. 10B-11B).

The highest amount of spurious causal estimates was obtained for intermediate level of the parameter *α*_*N*_, i.e. when the amount of mixed noise was similar to the amount of the unipolar signal in the measured time series, as in [13], [21]. Furthermore, when the mixed noise dominated (high *α*_*N*_), we observed a reduction in estimates for both GGC and tr-GGC, as shown in [21]. Finally, this simulation confirmed the positive effects associated with the use of time reversal testing to reduce spurious estimates due to the presence of additive noise [13], [20], [21].

### 3.4 Application of nonparametric GGC to Stokes and Purdon׳s example

We used the script *sim_nonparGGC_StokesPurdon.m* considering the case of nonparametric GGC (multitaper-based). This allows to address a recent claim of pitfall associated with the use of conditional GGC [14]. The problem originates from fitting separately full and reduced MVAR models, which introduces a bias-variance trade-off in the estimates that further depends on the model order. This problem has been already recognized in previous studies [16], [29], and several methods have been proposed as a solution [29], [30], [31], [32]. Nevertheless, since this pitfall hit the news in the field of causality analyses yet again, we used our script to provide a simple demonstration that the nonparametric methods based on spectral factorization can overcome this problem.

**Fig. 12:**
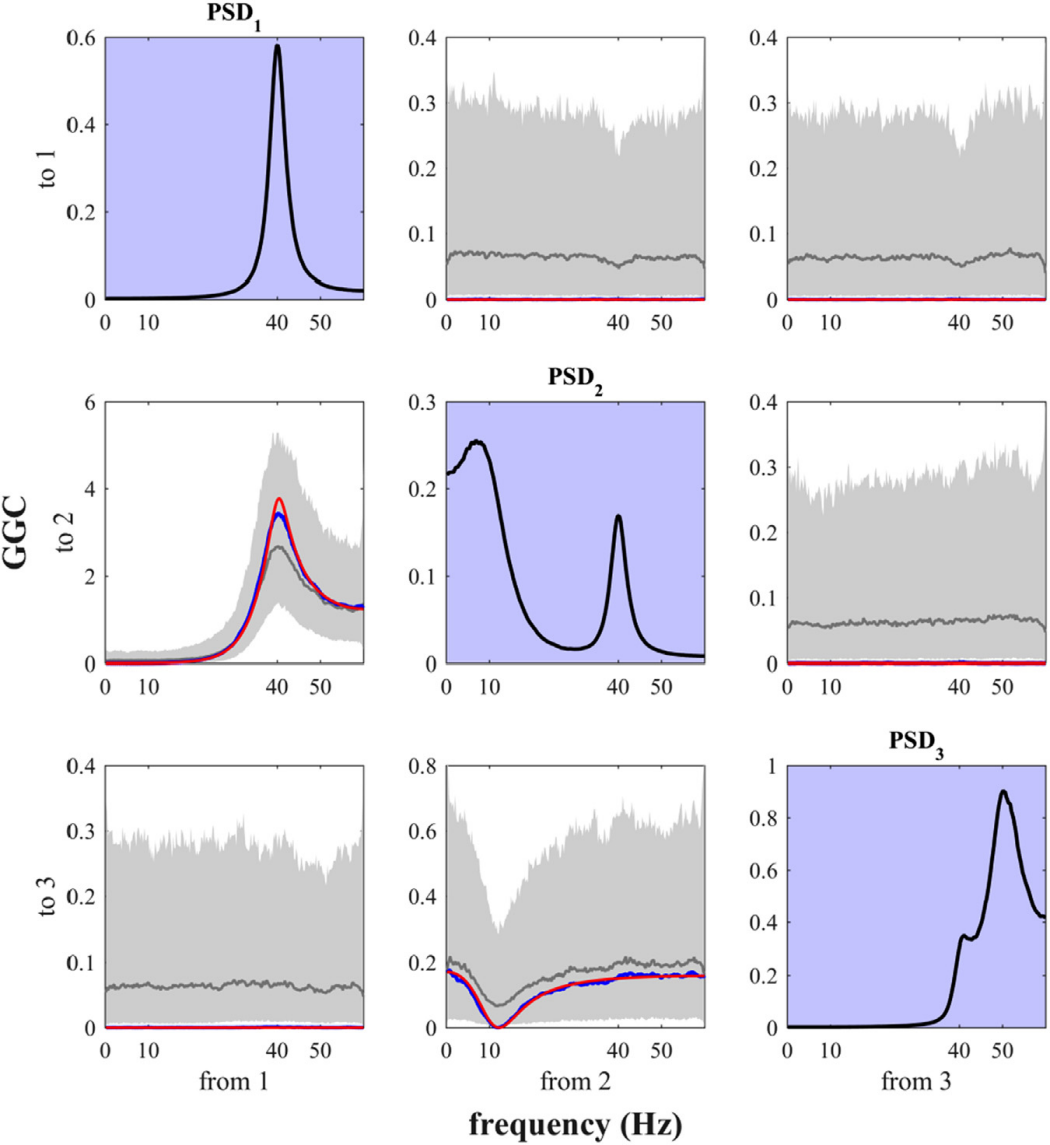
Conditional GGC and Power Spectral Densities (PSD) obtained with nonparametric multitaper-based method for the MVAR(3) three-nodes model, defined in [8]. Subplots on the diagonal show the PSD for each node (black line on purple background). The other subplots report true imposed GGC (red line), median GGC estimates across 1000 simulations (grey line) with corresponding 5th-95th percentiles (grey shading), and GGC estimates obtained by using all the realizations in the estimation (blue line), for each interaction from driver to receiver in the network.

We clearly observed unambiguous identification of the interactions imposed in the network from node 1 to node 2 and from node 2 to node 3, with respect to the estimates on edges with null interactions (Fig. 12). In particular by using all the realizations together in the estimation, the resulting GGC estimates reproduced very well the spectral profiles of true imposed values, confirming that the nonparametric (spectral factorization-based) method for GGC estimation [17], [18] recovers the underlying true network interactions, differently with respect to the parametric method used in [14].
