# Supplementary material for: Assessing the performance of Granger–Geweke causality: Benchmark dataset and simulation framework
Source: Data Brief. 2018 Oct 16;21:833–51. doi: 10.1016/j.dib.2018.10.034 (PMC6216071; doi:10.1016/j.dib.2018.10.034)
Supplement: Supplementary file 1 — Supplementary material [file mmc1.pdf]

UNIVERSITÉ DE FRIBOURG
UNIVERSITÄT FREIBURG

Mattia Federico Pagnotta, M.Sc.
University of Fribourg
Department of Psychology
Rue de Faucigny 2,
1700 Fribourg, Switzerland

Tel: +41 (0)26 300 7629
Email: mattia.pagnotta@unifr.ch

September 29th, 2018

Manuscript No.: DIB-D-18-01897
Title: Assessing the performance of Granger-Geweke causality: benchmark dataset and simulation framework
Journal Title: Data in Brief
Corresponding Author: Mr. Mattia Federico Pagnotta

Re: Conflict of Interest and Authorship Conformation Form

We declare that:

- All authors have participated in (a) conception and design, or analysis and interpretation of the data; (b) drafting the article or revising it critically for important intellectual content; and (c) approval of the final version.

- This manuscript has not been submitted to, nor is under review at, another journal or other publishing venue.

- The authors have no affiliation with any organization with a direct or indirect financial interest in the subject matter discussed in the manuscript.

On behalf of the authors,

Mattia F. Pagnotta
