# Supplementary material for: Assessing the performance of Granger–Geweke causality: Benchmark dataset and simulation framework
Source: Data Brief. 2018 Oct 16;21:833–51. doi: 10.1016/j.dib.2018.10.034 (PMC6216071; doi:10.1016/j.dib.2018.10.034)
Supplement: Supplementary file 2 — Supplementary material [file mmc2.zip › Benchmark dataset 10 rats/README.pdf]

Benchmark dataset: whisker-evoked SEPs from 10 rats

This dataset comprises one *.mat* file for each animal, for a total of 10. These files were created using MATLAB® (The MathWorks, Inc.).

Each file contains a structure named *RAT* with four fields:

- *data*: 3-dimensional matrix containing the epicranial SEPs (dimensions: time-points by number of channels by number of trials);
- *dimord*: string that specifies the dimensionality ordering of data;
- *times*: vector of time-points in milliseconds;
- *Fs*: scalar that reports the sampling rate in Hz.

A simple check on the data can be performed by plotting the event-related potentials specific for each rat. This can be done in MATLAB by using the following lines of code:

```
load(fullfile(your_directory, 'Benchmark data 10 rats\IC070523.mat'));  % load
file for first rat
figure
plot(RAT.times, mean(RAT.data,3));  % plot average across trials
ylabel('V (uV)');
xlabel('time (ms)');
```
