# Supplementary material for: Assessing the performance of Granger–Geweke causality: Benchmark dataset and simulation framework
Source: Data Brief. 2018 Oct 16;21:833–51. doi: 10.1016/j.dib.2018.10.034 (PMC6216071; doi:10.1016/j.dib.2018.10.034)
Supplement: Supplementary file 3 — Supplementary material [file mmc3.zip › nonparametricGGC_toolbox/README.pdf]

nonparametricGGC_toolbox

nonparametricGGC_toolbox. Copyright (©) 2018, Mattia F. Pagnotta.

This program is free software: you can redistribute it and/or modify it under the terms of the GNU General Public License as published by the Free Software Foundation, either version 3 of the License, or (at your option) any later version.
This program is distributed in the hope that it will be useful, but WITHOUT ANY WARRANTY; without even the implied warranty of MERCHANTABILITY or FITNESS FOR A PARTICULAR PURPOSE. See the GNU General Public License for more details.
You should have received a copy of the GNU General Public License along with this program. If not, see <https://www.gnu.org/licenses/>.

If you use nonparametricGGC_toolbox for a paper or talk please include the following references:

1) M.F. Pagnotta, M. Dhamala, G. Plomp, Benchmarking nonparametric Granger causality: robustness against downsampling and influence of spectral decomposition parameters, NeuroImage. 183 (2018) 478–494. https://doi.org/10.1016/j.neuroimage.2018.07.046
2) M. Dhamala, G. Rangarajan, M. Ding, Analyzing information flow in brain networks with nonparametric Granger causality, NeuroImage. 41 (2008) 354–362. https://doi.org/10.1016/j.neuroimage.2008.02.020.

If you want to report bugs or provide suggestions please send an e-mail to:
mattia.f.pagnotta@gmail.com (subject: nonparametricGGC_toolbox).

Nonparametric GGC estimation

The three files *compute_nonparGGC_multitaper.m*, *compute_nonparGGC_wavelet_Morlet.m*, and *compute_nonparGGC_wavelet_Paul.m* are functions implemented in MATLAB® (The MathWorks, Inc.), which allow estimating Granger–Geweke causality (GGC) [1] from multivariate data using nonparametric methods based on spectral factorization [2,3]. More specifically, the first function enables GGC estimation based on multitaper method [4]; while, the other two functions allow a straightforward estimation of time-varying nonparametric GGC using Morlet and Paul wavelet transforms [5], respectively. The spectral factorization, which is needed in nonparametric methods, is based on Wilson's

algorithm [6] and is performed through the function *wilson_sf.m*. Detailed comments about input/output of the functions and the different steps for GGC estimation are provided inside each script. For a more detailed description of nonparametric methods please refer to [2,3].

Simulation framework

The simulation framework comprises three MATLAB scripts:

- *sim_nonparGGC_CommonReference.m* relative to the common reference problem [7];
- *sim_nonparGGC_AdditiveNoise.m* relative to the problem of signal-to-noise ratio (SNR) imbalance between channels and the effects of additive noise [8];
- *sim_nonparGGC_StokesPurdon.m* relative to a recent claim of pitfall associated with the use of conditional GGC [9].

In each simulation, the function *compute_nonparGGC_multitaper.m* is used for estimating nonparametric GGC. Examples and detailed descriptions of the simulation framework are provided in the accompanied Data in Brief article.

References

[1] J. Geweke, Measurement of linear dependence and feedback between multiple time series, J. Am. Stat. Assoc. 77 (1982) 304–313. doi:10.2307/2287238.

[2] M. Dhamala, G. Rangarajan, M. Ding, Analyzing information flow in brain networks with nonparametric Granger causality, NeuroImage. 41 (2008) 354–362. doi:10.1016/j.neuroimage.2008.02.020.

[3] M.F. Pagnotta, M. Dhamala, G. Plomp, Benchmarking nonparametric Granger causality: Robustness against downsampling and influence of spectral decomposition parameters, NeuroImage. 183 (2018) 478–494. doi:10.1016/j.neuroimage.2018.07.046.

[4] D.J. Thomson, Spectrum estimation and harmonic analysis, Proc. IEEE. 70 (1982) 1055–1096. doi:10.1109/PROC.1982.12433.

[5] C. Torrence, G.P. Compo, A Practical Guide to Wavelet Analysis, Bull. Am. Meteorol. Soc. 79 (1998) 61–78. doi:10.1175/1520-0477(1998)079<0061:APGTWA>2.0.CO;2.

[6] G.T. Wilson, The factorization of matricial spectral densities, SIAM J. Appl. Math. 23 (1972) 420–426. doi:10.1137/0123044.

[7] A. Trongnetrpunya, B. Nandi, D. Kang, B. Kocsis, C.E. Schroeder, M. Ding, Assessing Granger Causality in Electrophysiological Data: Removing the Adverse Effects of Common Signals via Bipolar Derivations, Front. Syst. Neurosci. 9 (2016). doi:10.3389/fnsys.2015.00189.

[8] A.M. Bastos, J.-M. Schoffelen, A Tutorial Review of Functional Connectivity Analysis Methods and Their Interpretational Pitfalls, Front. Syst. Neurosci. 9 (2016). doi:10.3389/fnsys.2015.00175.

[9] P.A. Stokes, P.L. Purdon, A study of problems encountered in Granger causality analysis from a neuroscience perspective, Proc. Natl. Acad. Sci. 114 (2017) E7063–E7072. doi:10.1073/pnas.1704663114.
